# A Double-Blind, Randomized Placebo-Controlled Trial of Probiotic *Lactobacillus casei* Shirota in Stable Cirrhotic Patients

**DOI:** 10.3390/nu12061651

**Published:** 2020-06-02

**Authors:** Jane Macnaughtan, Francesco Figorilli, Elisabet García-López, Haw Lu, Helen Jones, Rohit Sawhney, Kaori Suzuki, Sarah Fairclough, Joanne Marsden, Alba Moratalla, I. Jane Cox, Linda Thomas, Nathan Davies, Roger Williams, Raj Mookerjee, Gavin Wright, Rajiv Jalan

**Affiliations:** 1 UCL Institute for Liver and Digestive Health, Division of Medicine, University College London, London NW3 2PF, UK; francesco.figorilli@gmail.com (F.F.); winstonluhaw@gmail.com (H.L.); helen.jones@ucl.ac.uk (H.J.); rohit.sawhney@austin.org.au (R.S.); info@albamoratalla.es (A.M.); nathan.davies@ucl.ac.uk (N.D.); r.mookerjee@ucl.ac.uk (R.M.); gavin.wright@btuh.nhs.uk (G.W.); r.jalan@ucl.ac.uk (R.J.); 2 Department of Hepatology, Royal Free Hospital, London NW3 2QG, UK; 3 Data Management Centre, European Foundation for the Study of Chronic Liver Failure (EF-CLIF), 08021 Barcelona, Spain; elisabet.garcia@efclif.com; 4 Yakult Europe B.V., 1332 EN Almere, The Netherlands; KSuzuki@yakulteurope.com (K.S.); drlvthomas@gmail.com (L.T.); 5 Mid and South Essex NHS Foundation Trust, Basildon & Thurrock University Hospitals NHS Foundation Trust, Basildon SS16 5NL, UK; Sarah.Fairclough@btuh.nhs.uk; 6 Department of Biochemistry, Bessemer Wing, King’s College Hospital, London SE5 9RS, UK; joannewillcox@aol.com; 7 Institute of Hepatology London, Foundation for Liver Research, London SE5 9NT, UK; j.cox@researchinliver.org.uk (I.J.C.); r.williams@researchinliver.org.uk (R.W.); 8 Faculty of Life Sciences & Medicine, King’s College London, London SE5 9RS, UK

**Keywords:** probiotic, cirrhosis, neutrophil, cytokine

## Abstract

Background: In cirrhosis, a pathological gut microbiome has been linked with immune dysfunction. A pilot study of probiotic Lactobacillus casei Shirota (LcS) in alcoholic cirrhosis demonstrated significant improvement in neutrophil function. This study aimed to evaluate the efficacy of LcS on neutrophil function and significant infection rates in patients with cirrhosis. Methods: 92 cirrhotic patients (Child–Pugh score ≤10) were randomized to receive LcS or placebo, three times daily for six months. Primary end-points were incidence of significant infection and neutrophil function. Secondary end-points were cytokine profile, endotoxin, bacterial DNA positivity, intestinal permeability and quality of life. Results: Rates of infection, decompensation or neutrophil function did not differ between placebo and probiotic groups. LcS significantly reduced plasma monocyte chemotactic protein-1 and, on subgroup analysis, plasma interleukin-1β (alcoholic cirrhosis), interleukin-17a and macrophage inflammatory protein-1β (non-alcoholic cirrhosis), compared with placebo. No significant differences in intestinal permeability, bacterial translocation or metabolomic profile were observed. Conclusion: LcS supplementation in patients with early cirrhosis is safe. Although no significant infections were observed in either group, LcS improved cytokine profile towards an anti-inflammatory phenotype, an effect which appears to be independent of bacterial translocation.

## 1. Introduction

Patients with cirrhosis exhibit a heightened susceptibility to infection [1]. The resultant systemic inflammatory response has been shown to predict poor outcome in acute decompensation and acute-on-chronic liver failure (ACLF). The CANONIC study demonstrated that patients with a previous history of early cirrhosis who developed ACLF had worse prognosis compared to patients with a history of decompensated disease, and this effect was more marked, with increasing leucocyte count, implicating the innate inflammatory response in pathogenesis [2].

Neutrophils constitute an important component of the innate inflammatory response. Neutrophil function has been shown to be disordered in cirrhosis, characterized by an increased resting burst and diminished neutrophil phagocytosis. This is associated with increased risk of infection, organ failure and mortality. Previous studies from our group have demonstrated that this neutrophil functional defect is possibly mediated by circulating humoral factors [3], including endotoxin. Ex vivo treatment of plasma from patients with anti-CD14 antibodies or with endotoxin removal columns prevented transference of susceptibility to normal neutrophils, implicating endotoxemia in pathogenesis. In keeping with this hypothesis, long-term intestinal decontamination with norfloxacin in cirrhotic patients reduces the incidence of infective episodes and complications [4].

Increased circulatory endotoxin concentrations in patients with cirrhosis are due to bacterial translocation from the gut. This is driven by a dysbiotic microbiota with a predominance of Gram-negative bacteria [5,6]. Increased intestinal permeability and diminished immune surveillance further contribute. Restoring homeostasis of the gut microbiota has the potential to decrease circulating endotoxin concentrations, leading to restoration of immune function in cirrhosis patients. Probiotics have been demonstrated to modulate the gut microbiota with positive effects on liver injury in experimental models of liver disease, dependent on the bacterial species used [7]. Probiotics have also been shown to decrease the severity of hepatic encephalopathy, hepatic venous pressure gradient, improve liver biochemistry and decrease the rate of infection after liver transplantation [8,9,10,11]. Several meta-analyses have already supported the use of probiotics in the prevention of infection in the general hospital population, but the exact mechanism is not fully understood [12,13,14].

A previous, pilot, open label study performed by our group evaluated the effects of *Lactobacillus casei* Shirota (LcS) in twelve patients with alcoholic cirrhosis [15]. Baseline neutrophil function showed a significantly lower phagocytic capacity in patients compared with controls, which normalized following four weeks of LcS therapy. This was associated with a significant reduction in tumor necrosis factor receptors 1 and 2 and interleukin-10 (IL-10) concentrations, providing proof of concept evidence that the functional phagocytic defect and the altered cytokine profile observed in cirrhosis could be restored with LcS.

The primary end-point of this study was to determine whether administration of LcS resulted in an improvement in neutrophil function and a reduction in the incidence of infection compared with placebo. Secondary end-points were to evaluate changes in gut barrier function (serum bacterial DNA positivity, intestinal permeability assays and urinary proton nuclear magnetic resonance (1H NMR) spectroscopy metabolic profiling, cytokine response and quality of life.

## 2. Materials and Methods

### 2.1. Patient Selection, Randomization and Study Outline

A double-blind, randomized and placebo-controlled study of LcS treatment with clinical, radiological and/or histological evidence of cirrhosis of any cause was conducted in two UK hospitals. The protocol conformed to the ethical guidelines of the 1975 Declaration of Helsinki and was approved by the joint University College London (UCL)/UCLH Committees on the Ethics of Human Research (ISCRCTN URL http://www.isrctn.com/ISRCTN62619436). Informed consent was obtained from all patients included in this study.

Inclusion criteria: Patients were aged between 18 and 78 years and were abstinent from alcohol for at least two weeks prior to the time of screening. Exclusion criteria: Child–Pugh score greater than 10; active infection; any antibiotic treatment within 7 days prior to enrollment, gastrointestinal haemorrhage within 2 weeks, use of immunomodulating agents within one month; use of proton pump inhibitors for the preceding two weeks; concomitant use of supplements (pre-, pro- or synbiotics) likely to influence the study; creatinine >150 mmol/L; hepatic encephalopathy II to IV; pancreatitis; other organ failure; hepatic or extrahepatic malignancy; pregnancy.

The patients were randomized (1:1) to receive a 65 mL bottle of LcS (6.5 × 10^9^ colony forming units (CFU)/bottle) or placebo (similar looking and tasting drink without bacteria) 3 times per day for 6 months (Yakult Europe). The randomization list was generated by an independent statistician at the University College London Biomedical Research Unit. Randomization was stratified for alcoholic and non-alcoholic aetiology of cirrhosis. Each participant was issued 45 bottles of the investigational product every two weeks for the duration of the study. Both investigators and participants were blinded to the intervention allocation. Compliance was measured by counting returned empty bottles. Non-compliance of greater than one month resulted in withdrawal from the study.

Clinical assessments, including routine haematological and biochemical tests, were performed at screening, days 0 and 14, and months 1, 3 and 6. Additional plasma and urine samples were collected and the intestinal permeability test was performed at the 0-, 1- and 6-month time-points.

### 2.2. Neutrophil Function Tests

Neutrophil isolation and coincubation were performed as described previously [2,14]. The Phagoburst and Phagotest kits (Orpegen Pharma, Heidelberg, Germany) were used in accordance with the manufacturer’s instructions. Cells were then coincubated in the dark at 4 °C with anti-CD16-(Phycoerythrin (PE)) and anti-CD11b-(Allophycocyanin (APC)-Cyanine-7(Cy7)) for 30 minutes. Samples were immediately analyzed by flow cytometry (BD LSR Fortessa, San Jose, CA, USA). Data were analyzed using FlowJo software (Ashland, OR, USA). Abnormal neutrophil function was defined as reactive oxygen species (ROS) production greater than 155% (in any of the following: Phosphate Buffered Saline (PBS), *Escherichia coli*, fLMP or phorbol 12-myristate 13-acetate (PMA) assays) or phagocytosis less than 42% as compared to neutrophils coincubated with healthy plasma. These thresholds were selected as they are known to be associated with increased mortality [3]. For further information, please refer to the Supplementary Materials.

### 2.3. Lactulose/Rhamnose Assay

Intestinal permeability was assessed by measuring lactulose/rhamnose ratios in urine collected after ingestion of a solution containing 5 g of lactulose, 1 g L-rhamnose and 0.2 g 3-O-methyl-D-glucose, reconstituted in 100 mL water (BCM Ltd, Nottingham, UK). The syrups were consumed following an overnight fast and baseline urine collection, and urine was collected for the subsequent 5 h. Urine samples were prepared for analysis by dilution in an 80:20 (v/v) mixture of acetonitrile:water containing internal standard 13C-xylose (CK-Gas Products Ltd, Newtown Unthank, UK). Samples were centrifuged and the supernatant transferred to a glass vial and injected onto a liquid chromatography-tandem mass spectrometer (LC-MS). The sugars were separated by high performance liquid chromatography on a Jasco LC-2000 series using an amino column in hydrophilic interaction liquid chromatography mode and quantitated by electrospray tandem mass spectrometry on an API 3200 LC-MS/MS system (Applied Biosystems, Foster City, CA, USA).

### 2.4. Markers of Bacterial Translocation

The chromogenic limulus amoebocyte lysate kinetic assay (Charles River Laboratories, Wilmington, MA, USA) was used according to the manufacturer’s instructions. Bacterial PCR was conducted on serum as previously described [16].

### 2.5. Plasma Cytokine Measurement

Plasma concentrations of IL-1, IL-2, IL-4, IL-6, IL-8, IL-10, IL-12, IL-17A, Interferon (IFN), Monocyte Chemotactic protein-1 (MCP-1), Macrophage Inflammatory Protein-1 (MIP-1) and Tumour Necrosis Factor α (TNFα) were measured in Ethylenediaminetetraacetic acid (EDTA) anticoagulated plasma using a Bio-Plex Pro human cytokine assay kit (Bio-Rad Laboratories Ltd., Watford, UK) and a Bio-Plex Magpix instrument (Bio-Rad Laboratories Ltd., Watford, UK), according to the manufacturer’s instructions.

### 2.6. Urinary Metabolic Profiling Using 1H NMR Spectroscopy

Urine was prepared for 1H NMR analysis using a standard protocol [17]. 1H NMR spectra were recorded as previously described [18]. 1H NMR spectra were processed using the Bruker AMIX data processing package and the KnowItAll Informatics System v17.1 (Bio-Rad, CA, USA). 1H NMR peaks in the range δ 0.50–9.50 ppm were analysed, excluding region δ 4.50–6.40 ppm to remove the residual water and urea signals. The urinary 1H NMR peaks were assigned with reference to the published literature [19].

The 1H NMR data were analyzed using principal component analysis (PCA) (KnowItAll Informatics System v17.1, Hercules, CA, USA). The data were subdivided into smaller regions of about 0.02 ppm using the intellibucketing option. All spectral regions were integrated, normalized to the sum of the total spectral integral and mean-centered prior to multivariate analysis. PCA was performed to highlight outliers and clustering. PCA was repeated according to particular subgroupings, including all points and with outliers excluded, and the class separation noted [20].

### 2.7. Statistical Analysis

Statistical analyses were performed by an independent statistician based at the European Foundation for the Study of Chronic Liver Failure using SAS v9.4 software (Marlow, Buckinghamshire, UK). Sample size calculation was based on the results of a prior study. The study sample size of 92 was estimated to provide 80% power to detect an increase in neutrophils able to undertake phagocytosis of more than 25%, with a 0.05 two-sided significance level, assuming a 20% dropout rate.

Two analysis populations were considered for analyses: the “as-treated (AT) population”, which consisted of all randomized patients who received at least one dose of LcS or placebo, and the “intention-to-treat (ITT)” population, which consisted of all randomized patients. Analysis of “alcoholic” and “non-alcoholic” subgroups in each of these populations was scheduled prospectively. Subgroup analysis of patients with baseline abnormal neutrophil function (defined as ROS production >55% or phagocytosis of <42%) was performed as an exploratory analysis.

All parameters related to neutrophil function, intestinal permeability assays, endotoxin, serum bacterial DNA positivity, plasma cytokine concentrations and urine 1H NMR spectroscopy, as well as clinical status during the study, laboratory parameters and quality of life assessment were analyzed in the full sets of patients. In addition, parameters of the neutrophil ROS production were analyzed in the subpopulation of patients with abnormal baseline neutrophil function. Cytokine concentrations and quality of life assessment were additionally analyzed in both the alcoholic cirrhotic cohort and the non-alcoholic cirrhotic cohort separately. All parameters were descriptively analyzed and compared at different time-points of evaluation.

Undetectable levels of cytokine parameters were assigned a value equal to the lower limit of detection. Undetectable levels of laboratory parameters were set to the midpoint from 0 to the lower limit of detection. Items of the 36-item Short Form Survey (SF-36) of quality of life were reduced to 8 scales by recoding and averaging according to the scoring rules of Rand Corporation (www.rand.org).

Discrete variables were described as counts (percentage) and continuous variables as mean (SD). Non-normally distributed variables were summarized by the median (interquartile range, IQR). In univariate statistical comparisons, the Chi-square test was used for categorical variables, whereas the Student t-test was used for normal continuous variables and the Mann–Whitney U test for continuous variables not normally distributed. In all statistical analyses, significance was set at *p* < 0.05.

## 3. Results

### 3.1. Patient Characteristics

**Figure 1:**
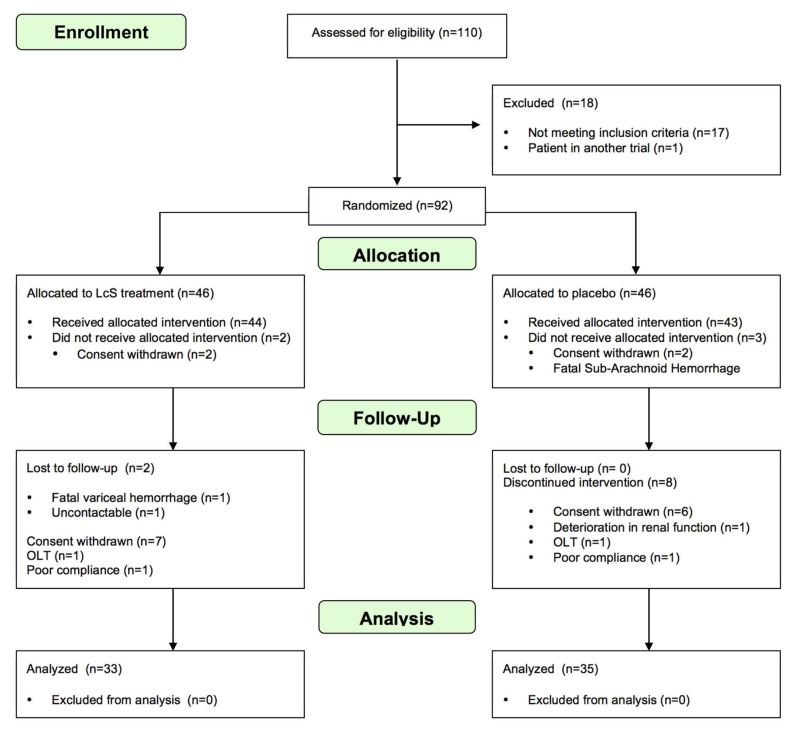
CONSORT flow diagram of the study [21].

Ninety-two patients were recruited into the study and randomized 1:1 to treatment with placebo or LcS. Placebo and treatment groups at initiation of treatment were well matched (Table 1). Most patients were Child–Pugh class A (placebo 88.1% vs. probiotic 83.3%). Indications for patient withdrawal or dropout are detailed in Figure 1. “As-treated” and “intention-to-treat” analysis was identical with regards to primary and secondary laboratory end-points.

### 3.2. Neutrophil Function

No significant differences in neutrophil ROS production or phagocytosis were observed between placebo and probiotic groups following six months of supplementation (Table 2A–C). However, in patients with abnormal baseline neutrophil function (Supplementary Table S3A,B), LcS therapy was associated with a significant improvement in phorbol 12-myristate 13-acetate (PMA)-induced neutrophil ROS production at month 6 compared to placebo control (geometric mean fluorescent intensity (GMFI), median (IQR): placebo 1168 (1014–1266) vs. LcS 1403 (1214–1821), *p* = 0.02) (Table 2B). No significant differences in constitutive, *Escherichia coli* (*E. coli*)-induced or N-formyl-methionyl-leucyl-phenylalanine (fMLP)-induced neutrophil ROS production were observed in the subgroup with baseline abnormal neutrophil function (Table 2B).

### 3.3. Infective Episodes and Decompensation Rates

No episodes of infection warranting hospitalization occurred in this study. Baseline frequency of infective episodes was low in both groups (<10%) and not significantly different between groups (Table 3A). A single episode of lower respiratory tract infection was observed in the placebo group at month 1. A single coryzal episode was observed in the treatment group at month 6 in contrast to two episodes in the placebo group.

Baseline frequency of decompensation episodes was low in both groups (<10%) with no statistically significant differences (Table 3B). No episodes of hepatic encephalopathy or hepatorenal syndrome were observed at months 1 and 6 in either treatment group. No significant difference between placebo and LcS groups was observed with regards to ascites, variceal haemorrhage or jaundice at months 1 and 6 of treatment.

No significant differences in full blood count, C-reactive protein, amylase, renal and liver biochemistry were observed at baseline or at month 6. Statistical significance was only observed between the placebo and LcS groups at month 1 with regards to haemoglobin and potassium. Neither of these differences was judged to be clinically significant (Supplementary Table S1).

### 3.4. Intestinal Permeability, Markers of Bacterial Translocation and Urinary Metabolic Profiling

Intestinal permeability was in the normal range in both groups and did not change significantly in either group (Table 4). No significant differences in plasma endotoxin concentrations or bacterial DNA positivity were observed at baseline, month 1 or month 6 between the two groups. At baseline, the frequency of bacterial DNA positivity was low at 10.0% and 8.1% in the placebo and LcS groups, respectively.

The urinary 1H NMR spectral profiles were compared from 59 subjects (all aetiologies) at baseline with 48 subjects at month 6 (all aetiologies). 1H NMR signals from isopropyl alcohol (IPA) were observed in 41 of 107 data sets, and the IPA region was excluded from the PCA analysis. Additional confounding peaks were observed from acetaminophen (7 of 107 data sets), ethanol (2 of 107 data sets) and glucose (7 of 107 data sets). No class separation was observed in the urinary metabolic profiles using PCA between placebo versus probiotic-treated groups, either at baseline or at six months, nor between baseline versus month 6 within the placebo grouping or within the probiotic grouping. Representative 1H NMR spectra are shown in Supplementary Figure S1.

### 3.5. Plasma Cytokine Concentrations

LcS was associated with a significant reduction in median plasma MCP-1 concentrations in the total AT population following six months of therapy (*p* = 0.02) (Table 5A). No significant differences in plasma IL-1β, IL-2, IL-4, IL-6, IL-8, IL-10, IL-1 p70, IL-17A, IFN, MIP-1β and TNFα concentrations were observed between the two groups at baseline, month 1 and month 6 (Table 5A). On subgroup analysis of the alcoholic patient cohort, LcS significantly lowered median plasma IL-1β (*p* = 0.04) and MCP-1 concentrations (*p* = 0.04) (Table 5B). On subgroup analysis of the non-alcoholic patient cohort, LcS significantly lowered IL-17A concentrations (*p* = 0.02) (Table 5C). MIP-1β concentrations were also significantly lowered at six months in the LcS group (*p* = 0.04).

### 3.6. Quality of Life Assessment

No significant differences in SF-36 scores were observed between placebo and LcS groups either on analysis of the total AT population (Supplementary Table S2A) or alcoholic/non-alcoholic cirrhosis subgroup analysis (Supplementary Table S2B,C).

## 4. Discussion

Intestinal dysbiosis is pathophysiologically linked to all complications of cirrhosis and implicated in immune dysfunction, resulting in heightened susceptibility to infection [22]. A reduction in autochthonous bacterial populations in stool is typically observed in cirrhosis with reciprocal expansions in pathobionts, including proteobacteria [23]. A previous small open label study evaluating patients with alcoholic cirrhosis demonstrated a significant improvement in neutrophil function after one month of supplementation with LcS [15]. This follow-up study was therefore conducted to validate these findings and determine whether similar effects could be observed in patients with less severe all cause cirrhosis and whether six months was more efficacious than one month of treatment.

During the study period, there was a low frequency of infectious episodes, with no episodes of infection warranting hospitalization in either group, a potential function of the predominance of Child–Pugh A class cirrhotics. This inevitably impacted on any potential efficacy signal from LcS. No treatment-related serious adverse effects were observed and no clinically significant alterations in laboratory parameters were observed (including amylase), demonstrating the favorable safety profile of LcS in cirrhotic patients.

Neutrophil function was not significantly different between treatment groups following six months of supplementation. Baseline neutrophil function was found to be normal in 41% of patients in the placebo-treated group and 37% in the LcS-treated group. Following a post-hoc analysis evaluating only patients with abnormal neutrophil function at baseline, six months of LcS supplementation was associated with significantly higher neutrophil PMA-induced ROS production compared to placebo control. PMA-induced ROS production is a measure of integrity of pathways downstream of protein kinase C, evaluating the capacity for ROS production and a marker of propensity to immune exhaustion. Previous studies have demonstrated that although patients with compensated cirrhosis may have normal or high ROS production, PMA-induced ROS production is defective [24]. Indeed, in a cohort of patients with alcoholic cirrhosis, a negative correlation was identified between PMA-induced neutrophil ROS production and alanine aminotransferase (ALT )levels [25].

**Figure 2:**
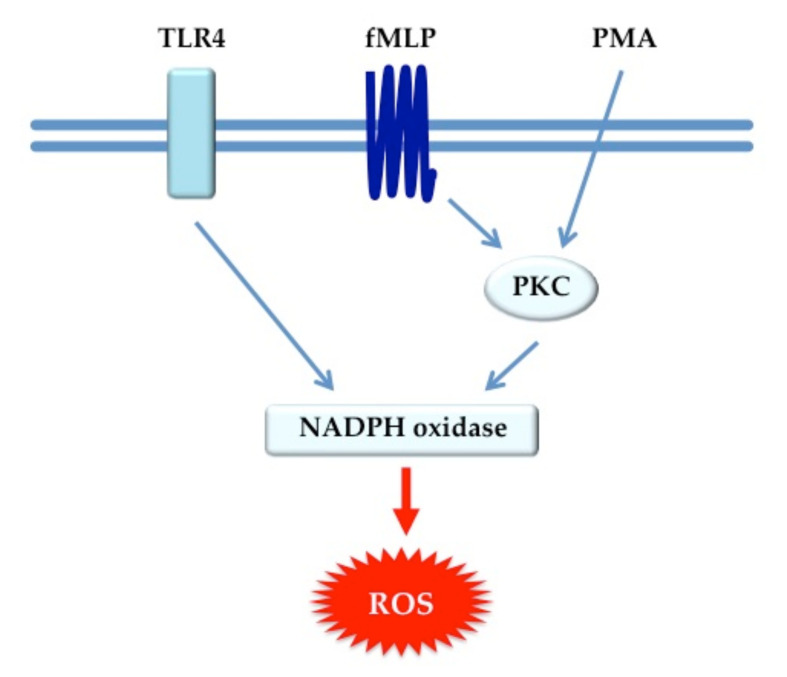
Intracellular signaling pathways of neutrophil ROS production.

The absence of significant effects of LcS on constitutive, *E. coli*- and fMLP-induced ROS production and phagocytosis with LcS compared to placebo suggests that LcS may be acting independently of toll-like receptor (TLR-4) or fMLP neutrophil pathways but this needs to be evaluated in future studies (Figure 2). Whilst both groups exhibited heightened constitutive and fMLP-induced neutrophil ROS production indicative of a primed state, no significant differences were observed between the two groups at months 1 and 6 post-treatment. 

A significant reduction in plasma concentrations of MCP-1 was observed following six months of LcS compared to placebo. MCP-1 is primarily secreted by monocyte, macrophages and dendritic cells and is a chemotactic factor for monocytes. MCP-1 concentrations have been shown to be elevated in patients with spontaneous bacterial peritonitis and resolve with resolution of the infection [26]. Polymorphisms of the MCP-1 gene confer differential susceptibility to spontaneous bacterial peritonitis, and its concentrations can predict survival in these patients [27]. A significant reduction in plasma MIP-1β was observed in patients with non-alcoholic cirrhosis following six months of treatment with LcS. MIP-1β is produced by macrophages in response to stimulation with bacterial endotoxin and exhibits proinflammatory effects. Previous studies have demonstrated significant increases in plasma concentrations in patients with ACLF [28]. A significant reduction in IL-17A concentrations was observed in patients receiving six months of LcS therapy in the non-alcoholic patients compared to placebo. Obesity and non-alcoholic fatty liver disease (NAFLD) have been associated with an increase in IL-17A, which is produced in the intestinal mucosa, and its levels are modulated by microbiome composition [29,30]. Germ-free mice have no intestinal T helper-17 cells [31]. Its role in the pathogenesis of liver disease is well established in multiple mouse models of liver injury. IL-17A has been shown to act directly on Kupffer cells, resulting in increased IL-6, TNFα and Transforming Growth Factor β (TGFβ) production [32]. IL-1β concentrations were found to be significantly lowered with LcS treatment in the alcoholic cirrhotic subgroup compared to placebo. IL-1β has an established role in alcoholic steatohepatitis, and attenuation of liver injury is observed with IL-1R^-/-^ mice and treatment with an IL-1R antagonist [33]. It is involved in all inflammatory processes in the liver, induces steatosis, activates stellate cells and impairs intestinal barrier function [34].

No significant differences in intestinal permeability were observed with LcS; indeed, all the measured values were within normal range, perhaps a reflection of the predominance of Child–Pugh A patients in this study. In keeping with this observation, plasma endotoxin and bacterial DNA positivity rates were also very low. Previous studies have demonstrated progressive increases in intestinal permeability with advancing disease, in particular with increasing portal hypertension and active alcoholism [35]. The patients included in the present study had stable cirrhosis and were abstinent from alcohol for at least two weeks prior to enrollment, which may account for the findings. Urinary metabolomic profile was similar between the groups both at baseline and follow up, suggesting that changes in metabolism do not account for the beneficial effects of LcS. In keeping with these observations, a randomized, controlled study of LcS in patients with NAFLD did not affect the composition of the microbiome [36].

Rodent models of NAFLD have demonstrated that LcS supplementation is associated with attenuation in liver injury mediated via toll-like receptor (TLR-4)-related pathways [37]. Horvath et al. [38] demonstrated a significant but subclinical increase in constitutive neutrophil ROS production with supplementation of a multispecies probiotic for six months in a placebo-controlled study of stable outpatient cirrhotic patients. *E. coli*-/fMLP- or PMA-induced ROS production was not described and no impact on cytokine profile was observed. Other previous studies evaluating efficacy of probiotic therapy in cirrhosis have focused primarily on patients with more advanced disease than represented in this study, most markedly with encephalopathy. Dhiman et al. [39] observed a significant improvement in features of the Systemic Inflammatory Response Syndrome (SIRS), Child–Pugh score, IL-6 and TNFα concentrations. In this group, however, more than 60% of patients had Child–Pugh C and >30% patients had Child–Pugh B cirrhosis. No evaluation of neutrophil function was performed. Bajaj et al. [40] observed a significant decrease in TNFα and endotoxin concentrations with eight weeks of VSL#3 probiotic therapy in a study of 30 patients with minimal hepatic encephalopathy. In cirrhotic patients, the use of probiotics has been best studied in those with hepatic encephalopathy, and most studies suffer from similar problems of the use of multiple probiotics, concomitant use of prebiotics and other methodologic issues. The issues led a recent Cochrane meta-analysis to conclude that the cumulative data do not confirm that probiotics are better than lactulose for hepatic encephalopathy because the quality of the available evidence is very low. The results of our rigorously performed clinical trial has not confirmed any clinically meaningful effect of LcS for the treatment of patients with well-compensated cirrhosis.

Limitations of this study include the use of an ex vivo method in which neutrophil function was assessed following coincubation of healthy volunteers with patient plasma. This methodology has been validated in previous studies by Tritto et al. [41] and Stadlbauer et al. [15] in similar patient populations. It is, however, a more indirect method of assessing neutrophil function with propensity for disease-specific signals. Conversely, it does measure the cellular defect conferred by patient plasma and highlights how rapidly humoral factors, such as endotoxin, can induce pathological changes. The age range 18–78 was selected to be similar to that of the proof of concept data and to be representative of the target patient cohort. Studies have now demonstrated differences in microbiome composition between populations of different ages, including expansion of colonic Bacteroidetes populations [42], and may represent one factor contributing to the heterogeneity in response. The groups in this study, however, were well matched for age.

In conclusion, the results of this double-blind, randomized, placebo-controlled study show that the probiotic LcS is safe but had no significant effect on rates of significant infection, decompensation episodes or neutrophil function on scheduled statistical analysis in stable cirrhotic patients. A potential effect on the incidence of significant infections could not be detected due to low frequency in both groups. Favorable effects on cytokine profile were observed which appeared to be independent of effects on bacterial translocation. This may translate into a clinically measurable effect in a more advanced patient cohort but currently LcS supplementation cannot be recommended as a therapeutic strategy for stable cirrhotic patients. 

## Figures and Tables

**Table 1:** Patient characteristics at baseline.

	Total	Placebo	LcS	*p* Value
*n*	87	43	44	
Age (Mean ± SD)	57.15 ± 8.83	58.16 ± 9.18	56.16 ± 8.47	0.29
Male (*n* (%))	62 (71.26%)	30 (69.77%)	32 (72.73%)	0.76
Etiology of Cirrhosis (*n* (%))				
Alcohol	45 (51.72%)	24 (55.81%)	21 (47.73%)	0.45
Hepatitis B	6 (6.90%)	2 (4.65%)	4 (9.09%)	0.41
Hepatitis C	18 (20.69%)	12 (27.91%)	6 (13.64%)	0.10
NASH	11 (12.64%)	3 (6.98%)	8 (18.18%)	0.12
Other	7 (8.05%)	2 (4.65%)	5 (11.36%)	0.25
MELD (Mean ± SD)	9.02 ± 2.80	8.88 ± 2.38	9.15 ± 3.17	0.66
Child Pugh Class (*n* (%))				
A	72 (85.71%)	37 (88.10%)	35 (83.33%)	0.56
B	11 (13.10%)	5 (11.90%)	6 (14.29%)	
C	1 (1.19%)	0	1 (2.38%)	
History of decompensation event (*n* (%))				
Hepatic encephalopathy	8 (9.30%)	5 (11.63%)	3 (6.98%)	0.46
Ascites	33 (37.93%)	19 (44.19%)	14 (31.82%)	0.23
Jaundice	19 (22.09%)	10 (23.26%)	9 (20.93%)	0.79
Variceal Hemorrhage	39 (44.83%)	18 (41.86%)	21 (47.73%)	0.58

**Table 2:** Measures of neutrophil function.

**A**		**Time Point**	**Placebo (GMFI) (Median (IQR))**	**LcS (GMFI) (Median (IQR))**	***p*** **-Value**
	Constitutive ROS	Day 0	35.90 (25.50–51.10)	35.80 (26.65–48.70)	0.75
		Month 1	36.35 (25.75–64.15)	33.70 (27.00–47.60)	0.85
		Month 6	39.20 (26.80–71.00)	36.70 (29.20–57.00)	0.87
	E.coli-induced ROS	Day 0	760.00 (498.00–1031.00)	778.50 (496.50–1177.00)	0.83
		Month 1	744.50 (505.50–1073.50)	706.00 (526.00–1010.00)	0.97
		Month 6	604.00 (363.00–965.00)	719.00 (540.00–1049.00)	0.26
	fMLP-induced ROS	Day 0	76.40 (58.90–98.60)	92.05 (59.75–127.50)	0.22
		Month 1	76.35 (51.30–133.50)	88.10 (49.10–129.00)	0.64
		Month 6	85.80 (52.00–140.00)	86.85 (64.80–147.00)	0.68
	PMA-induced ROS	Day 0	1344.00 (1022.00–1857.00)	1574.00 (1175.00–2540.00)	0.19
		Month 1	1504.00 (1112.00–2401.00)	1490.00 (1219.00–2660.00)	0.19
		Month 6	1207.00 (1014.00–1837.00)	1493.00 (1228.50–2049.50)	0.07
**B**		**Time Point**	**Placebo (GMFI) (Median (IQR))**	**LcS (GMFI) (Median (IQR))**	***p*** **-Value**
	Constitutive ROS	Day 0	45.10 (35.20–74.50)	44.10 (31.60–81.50)	0.91
		Month 1	50.80 (30.30–83.70)	42.80 (30.90–101.35)	0.89
		Month 6	56.20 (35.30–72.40)	39.30 (29.20–84.20)	0.69
	E. coli-induced ROS	Day 0	567.00 (316.00–1023.00)	766.00 (513.00–1093.00)	0.16
		Month 1	553.00 (332.00–754.00)	669.00 (507.50–1066.50)	0.29
		Month 6	506.00 (287.00–687.00)	731.00 (516.00–1067.00)	0.06
	fMLP-induced ROS	Day 0	85.50 (65.05–131.50)	123.00 (92.00–146.00)	0.23
		Month 1	86.20 (57.40–149.00)	100.80 (73.50–174.50)	0.56
		Month 6	99.10 (73.00–180.00)	95.30 (65.90–149.00)	0.73
	PMA-induced ROS	Day 0	1149.00 (833.50–1654.50)	1460.00 (1179.00–1937.00)	0.07
		Month 1	1170.00 (925.00–2101.00)	1390.00 (1150.00–2043.50)	0.25
		Month 6	1168.00 (1014.00–1266.00)	1403.00 (1214.00–1821.00)	0.02
**C**		**Time Point**	**Placebo (GMFI) (Median (IQR))**	**LcS (GMFI) (Median (IQR))**	***p*** **-Value**
	Phagocytosis	Day 0	1556.00 (1070.00–1903.00)	1737.00 (1246.50–2124.50)	0.32
		Month 1	1562.00 (1042.50–1863.50)	1782.00 (1405.00–2302.00)	0.25
		Month 6	1433.00 (866.00–1985.00)	1626.50 (1062.00–1898.00)	0.62

Number of patients in placebo, *Lactobacillus casei* Shirota (LcS)-treated and total patient groups with abnormal neutrophil phosphate buffered saline (PBS), *Escherichia coli*, N-formyl-methionyl-leucyl-phenylalanine (fMLP), phorbol 12-myristate 13-acetate (PMA)-induced reactive oxidant species (ROS) production and phagocytosis. (A) Neutrophil reactive oxygen species (ROS) production in patients receiving placebo and LcS supplementation at day 0, month 1 and month 6 expressed as geometric mean fluorescent intensity (GMFI). (B) Neutrophil ROS production in a subpopulation of patients with abnormal neutrophil function at baseline receiving placebo and probiotic supplementation at day 0, month 1 and month 6 expressed as GMFI. (C) Neutrophil phagocytosis in patients receiving placebo and probiotic supplementation at day 0, month 1 and month 6 expressed as GMFI.

**Table 3:** Clinical episodes during study.

**A**		**Placebo (%)**			**LcS (%)**		
		**Day 0**	**Month 1**	**Month 6**	**Day 0**	**Month 1**	**Month 6**
	Spontaneous Bacterial Peritonitis	0	0	0	0	0	0
	Lower Respiratory Tract Infection	2.33	2.44	0	0	0	0
	Urinary Tract Infection	0	0	0	2.27	0	0
	Gastroenteritis	2.33	0	0	0	0	0
	Occult sepsis	0	0	0	0	0	0
	Other	6.98	2.44	5.72	0	0	3.23
**B**		**Placebo (%)**			**LcS (%)**		
		**Day 0**	**Month 1**	**Month 6**	**Day 0**	**Month 1**	**Month 6**
	Ascites	2.33	4.86	5.71	6.82	5.13	6.45
	Variceal Hemorrhage	0	0	0	0	1.27	1.52
	Jaundice	2.33	0	0	4.55	2.56	0
	Hepatic Encephalopathy	0	0	0	2.27	0	0
	Hepatorenal Syndrome	0	0	0	2.27	0	0

(A) Infective episodes during study. (B) Decompensation episodes during study.

**Table 4:** Markers of intestinal permeability and bacterial translocation.

Assay	Time Point	Placebo (*n*, Median (IQR))	LcS (*n*, Median (IQR))	*p*-Value
**Lactulose:Rhamnose Ratio**	Day 0	31, 0.03 (0.02–0.05)	30, 0.03 (0.02–0.04)	0.54
	Month 1	24, 0.03 (0.02–0.04)	21, 0.03 (0.02–0.04)	0.99
	Month 6	22, 0.03 (0.03–0.03)	18, 0.03 (0.02–0.05)	0.76
**Endotoxin (EU/mL)**	Day 0	28, 0.04 (0.01–0.06)	28, 0.04 (0.01–0.05)	0.87
	Month 1	32, 0.06 (0.02–0.14)	28, 0.05 (0.01–0.10)	0.27
	Month 6	23, 0.09 (0.01–0.17)	21, 0.04 (0.01–0.10)	0.5
		**Placebo (*n*, %)**	**LcS (*n*, %)**	
**Bacterial DNA Positivity**	Day 0	4 (10.00)	3 (8.11)	1
	Month 1	5 (12.82)	3 (7.89)	0.71
	Month 6	4 (12.90)	4 (13.33)	1

Intestinal permeability (as evidenced by the 5 h urinary excretion of lactulose and rhamnose), venous endotoxin concentrations and bacterial DNA positivity in patients receiving placebo and probiotic supplementation at day 0, month 1 and month 6.

**Table 5:** Plasma cytokine concentrations.

**A**	**Cytokine**		**Placebo (pg/mL) (Median (IQR))**	**LcS (pg/mL) (Median (IQR))**	***p*-Value**
	IL-1β	Day 0	0.31 (0.26–0.51)	0.41 (0.20–0.67)	0.65
		Month 1	0.31 (0.22–0.51)	0.26 (0.12–0.63)	0.65
		Month 6	0.34 (0.26–0.89	0.39 (0.09–0.73)	0.2
	IL-2	Day 0	1.14 (1.07–1.17)	1.14 (1.07–1.17)	0.8
		Month 1	1.14 (1.07–1.17)	1.17 (1.07–1.17)	0.87
		Month 6	1.14 (1.07–1.36)	1.14 (1.07–1.17)	0.86
	IL-4	Day 0	0.25 (0.14–0.33)	0.28 (0.19–0.31)	0.89
		Month 1	0.28 (0.19–0.31)	0.25 (0.19–0.31)	0.68
		Month 6	0.25 (0.14–0.59)	0.25 (0.16–0.30)	0.94
	IL-6	Day 0	2.31 (1.19–4.53)	2.31 (1.35–3.57)	0.54
		Month 1	2.31 (1.89–4.41)	2.28 (1.55–3.41)	0.46
		Month 6	2.46 (1.47–4.67)	2.31 (0.97–2.58)	0.12
	IL-8	Day 0	4.77 (3.22–8.50)	4.96 (3.33–7.20)	0.86
		Month 1	4.72 (2.68–7.48)	3.63 (2.02–6.95)	0.27
		Month 6	4.40 (3.11–7.58)	3.54 (2.55–6.39)	0.11
	IL-10	Day 0	1.71 (0.81–2.22)	1.72 (0.60–2.44)	0.72
		Month 1	2.15 (0.89–3.24)	2.15 (0.56–4.80)	0.85
		Month 6	2.00 (0.89–3.84)	2.15 (0.94–3.26)	0.75
	IL12_p70	Day 0	2.19 (1.11–4.74)	1.77 (1.23–4.10)	0.73
		Month 1	2.68 (1.46–5.00)	1.94 (1.00–5.14)	0.25
		Month 6	2.52 (1.47–5.80)	2.17 (0.88–2.96)	0.06
	IL-17A	Day 0	4.33 (1.63–8.75)	3.83 (1.99–5.99)	0.31
		Month 1	4.90 (1.99–11.01)	4.33 (1.99–6.99)	0.81
		Month 6	4.14 (1.74–15.78)	2.29 (1.87–6.36)	0.21
	IFN-γ	Day 0	21.87 (12.32 407.11)	25.94 (19.33–79.97)	0.98
		Month 1	21.87 (11.52–54.25)	52.20 (21.87–407.11)	0.11
		Month 6	21.87 (15.18–92.89)	21.87 (15.82-107.54)	0.85
	MCP-1	Day 0	11.09 (5.18–18.42)	12.22 (6.19–18.42)	0.36
		Month 1	11.18 (5.18–21.76)	7.60 (6.19–24.12)	0.96
		Month 6	13.70 (7.60–22.50)	8.11 (4.58–13.75)	0.02
	MIP-1b	Day 0	21.76 (17.57–32.49)	26.25 (18.98–33.41)	0.44
		Month 1	27.47 (18.64–40.21)	23.59 (15.27–29.98)	0.08
		Month 6	29.69 (22.84–36.26)	22.63 (17.16–32.66)	0.19
	TNF-α	Day 0	3.98 (1.59–8.98)	4.35 (1.59–7.66)	0.79
		Month 1	4.35 (2.11–9.66)	3.57 (1.18–13.10)	0.35
		Month 6	4.35 (2.91–9.93)	4.35 (1.18–12.92)	0.77
**B**	**Cytokine**		**Placebo (pg/mL) (Median (IQR))**	**LcS (pg/mL) (Median (IQR))**	***p*** **-Value**
	IL-1β	Day 0	0.29 (0.24–0.49)	0.26 (0.17–0.57)	0.47
		Month 1	0.26 (0.16–0.34)	0.22 (0.12–0.63)	0.88
		Month 6	0.41 (0.31–0.89)	0.14 (0.07–0.70)	0.04
	IL-2	Day 0	1.14 (1.07–1.17)	1.17 (1.07–1.17)	0.22
		Month 1	1.15 (1.14–1.38)	1.17 (1.14–1.17)	0.96
		Month 6	1.14 (1.07–1.50)	1.15 (1.07–1.17)	0.70
	IL-4	Day 0	0.25 (0.14–0.30)	0.19 (0.12–0.28)	0.27
		Month 1	0.25 (0.15–0.28)	0.25 (0.25–0.42)	0.34
		Month 6	0.28 (0.14–0.60)	0.25 (0.14–0.28)	0.57
	IL-6	Day 0	2.31 (0.97–5.89)	2.31 (1.43–3.73)	0.73
		Month 1	2.31 (2.15–6.38)	2.31 (1.62–3.41)	0.29
		Month 6	2.84 (1.90–8.39)	2.34 (1.20–2.78)	0.17
	IL-8	Day 0	4.72 (2.63–9.21)	5.80 (3.44–9.22)	0.57
		Month 1	4.62 (2.47–8.18)	4.59 (2.79–8.77)	0.90
		Month 6	4.96 (3.84–7.58)	4.03 (2.67–6.85)	0.45
	IL-10	Day 0	1.85 (0.99–2.22)	1.60 (0.60–2.22)	0.55
		Month 1	1.86 (0.58–3.18)	2.15 (0.56–4.80)	0.84
		Month 6	2.22 (1.72–4.01)	2.15 (0.94–4.29)	0.89
	IL12_p70	Day 0	2.51 (1.00–5.08)	1.70 (1.23–3.34)	0.72
		Month 1	3.15 (1.24–10.37)	2.21 (1.23–4.40)	0.27
		Month 6	3.34 (2.15–13.47)	2.29 (1.23–4.36)	0.12
	IL-17A	Day 0	3.42 (1.63–8.84)	3.96 (1.18–5.99)	0.67
		Month 1	4.25 (1.99–16.93)	4.98 (1.99–6.99)	1.00
		Month 6	1.99 (1.74–18.27)	3.60 (1.81–7.66)	0.87
	IFN-γ	Day 0	20.60 (7.38–40.99)	21.87 (11.52–407.11)	0.58
		Month 1	21.87 (11.09–407.11)	65.40 (21.87–407.11)	0.37
		Month 6	21.87 (19.33–50.27)	28.17 (14.67–273.81)	0.58
	MCP-1	Day 0	9.48 (4.35–15.71)	10.11 (6.19–15.83)	0.66
		Month 1	11.51 (4.96–20.92)	6.28 (6.19–25.31)	0.63
		Month 6	16.70 (9.11–24.19)	6.19 (4.58–13.36)	0.04
	MIP-1b	Day 0	21.58 (18.16–34.70)	26.25 (20.76–31.89)	0.51
		Month 1	27.88 (19.01–40.50)	25.42 (17.62–31.27)	0.37
		Month 6	24.75 (20.33–33.59)	28.71 (19.35–35.35)	0.47
	TNF-α	Day 0	3.45 (1.18–6.66)	2.91 (1.59–6.74)	0.84
		Month 1	3.30 (1.59–4.91)	4.35 (2.12–13.10)	0.60
		Month 6	4.35 (3.06–9.66)	4.35 (1.18–17.42)	0.91
**C**	**Cytokine**		**Placebo (pg/mL) (Median (IQR))**	**LcS (pg/mL) (Median (IQR))**	***p*** **-Value**
	IL-1β	Day 0	0.34 (0.26–0.52)	0.58 (0.46–0.94)	0.16
		Month 1	0.36 (0.31–0.78)	0.33 (0.14–0.62)	0.49
		Month 6	0.31 (0.17–0.52)	0.53 (0.26–0.73)	0.54
	IL-2	Day 0	1.17 (1.14–1.17)	1.14 (1.10–1.17)	0.27
		Month 1	1.14 (1.07–1.17)	1.14 (1.07–1.17)	0.90
		Month 6	1.17 (1.07–1.17)	1.14 (1.10–1.15)	0.42
	IL-4	Day 0	0.28 (0.14–0.36)	0.30 (0.28–0.52)	0.20
		Month 1	0.28 (0.24–0.36)	0.25 (0.12–0.30)	0.16
		Month 6	0.22 (0.14–0.28)	0.28 (0.21–0.34)	0.49
	IL-6	Day 0	2.31 (1.62–3.33)	1.70 (1.27–3.45)	0.48
		Month 1	2.14 (1.83–3.09)	2.14 (0.71–3.04)	0.93
		Month 6	2.31 (1.20–3.97)	1.82 (0.82–2.45)	0.36
	IL-8	Day 0	4.77 (3.63–6.24)	3.85 (2.26–4.63)	0.17
		Month 1	4.83 (2.68–6.82)	2.82 (1.57–4.77)	0.06
		Month 6	3.92 (3.00–6.02)	2.96 (1.88–4.21)	0.15
	IL-10	Day 0	1.69 (0.72–2.22)	2.07 (0.72–2.49)	0.93
		Month 1	2.22 (1.15–3.24)	2.15 (0.98–4.22)	0.83
		Month 6	1.15 (0.87–3.54)	2.07 (0.73–2.70)	0.98
	IL12_p70	Day 0	1.94 (1.23–2.68)	2.81 (0.63–4.49)	1.00
		Month 1	2.68 (1.46–4.49)	1.68 (0.66–5.53)	0.68
		Month 6	1.70 (1.47–4.74)	1.65 (0.59–2.62)	0.34
	IL-17A	Day 0	5.80 (1.99–8.75)	3.18 (1.99–6.00)	0.28
		Month 1	5.25 (1.99–9.12)	3.78 (1.99–7.09)	0.82
		Month 6	6.91 (2.25–9.83)	1.99 (1.87–2.64)	0.02
	IFN-γ	Day 0	66.07 (21.87–407.11)	27.54 (21.87–59.62)	0.36
		Month 1	21.87 (11.52–47.15)	22.41 (20.27–407.11)	0.27
		Month 6	49.69 (12.32–407.11)	21.87 (15.82–41.06)	0.50
	MCP-1	Day 0	13.14 (6.19–18.42)	15.83 (10.29–25.70)	0.39
		Month 1	11.18 (5.90–21.76)	7.60 (4.29–13.93)	0.59
		Month 6	12.77 (7.60–17.57)	9.61 (4.50–14.50)	0.31
	MIP-1b	Day 0	21.92 (17.57–28.10)	24.14 (18.16–33.47)	0.79
		Month 1	27.47 (17.38–40.21)	21.82 (14.19–24.41)	0.06
		Month 6	33.84 (29.85–36.82)	18.35 (16.28–22.63)	0.04
	TNF-α	Day 0	3.98 (2.25–11.72)	4.63 (4.30–13.65)	0.38
		Month 1	6.57 (3.52–10.38)	2.68 (0.96–12.58)	0.14
		Month 6	4.35 (2.91–9.93)	4.17 (1.65–10.40)	0.71

Plasma cytokine concentrations in patients receiving placebo and probiotic supplementation at day 0, month 1 and month 6. (A) Total population. (B) Alcoholic cirrhosis subgroup. (C) Non-alcoholic cirrhosis subgroup.

## Supplementary Materials

The following are available online at https://www.mdpi.com/2072-6643/12/6/1651/s1, Figure S1: Illustrative urinary 1H NMR spectra, Table S1: Laboratory parameters in placebo and LcS-treated patient populations, Table S2A: Quality of Life Assessment using the SF-36 questionnaire (total population), Table S2B: Quality of Life Assessment using the SF-36 questionnaire (alcoholic sub-population), Table S2C: Quality of Life Assessment using the SF-36 questionnaire (non-alcoholic sub-population), Table S3A: Number of abnormal neutrophil tests at baseline in placebo, LcS-treated and total patient groups, Table S3B: Number of patients in placebo, LcS-treated and total patient groups with abnormal neutrophil PBS-, E.coli, fMLP, PMA-induced Reactive Oxidant Species (ROS) production and phagocytosis, Table S4: Gastrointestinal Adverse Event Profile.
