# Supplementary material for: A Double-Blind, Randomized Placebo-Controlled Trial of Probiotic Lactobacillus casei Shirota in Stable Cirrhotic Patients
Source: Nutrients. 2020 Jun 2;12(6):1651. doi: 10.3390/nu12061651 (PMC7352321; doi:10.3390/nu12061651)
Supplement: Supplementary file 1 [file nutrients-12-01651-s001.pdf]

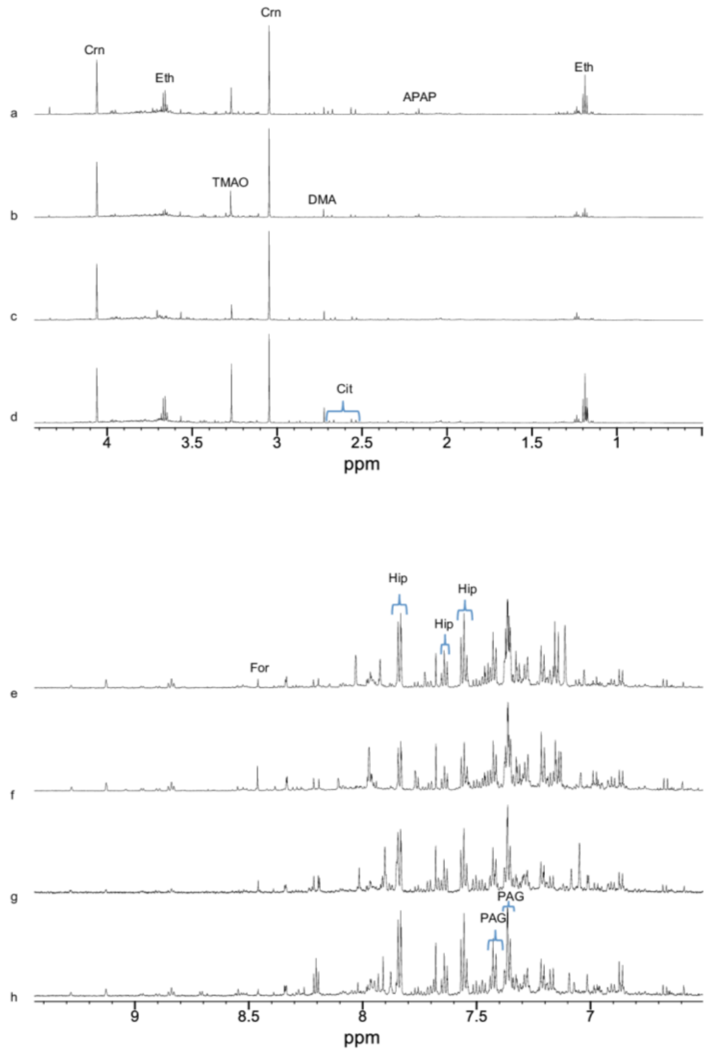

Figure S1. Illustrative urinary proton NMR spectra from: a male with alcoholic cirrhosis, Child Pugh A, MELD 9 (a,e) at day 0 and (b,f) six month after placebo supplementation; and male with alcoholic cirrhosis, Child Pugh A, MELD 12 (c,g) at baseline and (d,h) six month after LcS supplementation. Each NMR spectrum is scaled independently and (a-d) shows the aliphatic region 0.5-4.5 ppm and (e-h) displays the aromatic region 6.5-9.5 ppm.

Table S1. Laboratory parameters in placebo and LcS-treated patient populations.

Laboratory Parameter		Placebo (Median(IQR))	LcS (Median(IQR))	p value
Haemoglobin (g/dL)	Day 0	14.30 (13.00- 15.10)	13.60 (12.40- 14.70)	0.16
	Month 1	14.30 (13.30- 15.10)	13.55 (12.15- 14.55)	0.08
	Month 6	14.20 (13.00- 15.00)	13.45 (12.20- 15.10)	0.36
Leucocyte Count (10^9/L)	Day 0	5.80 (4.72- 7.37)	5.88 (4.70- 7.30)	0.88
	Month 1	5.60 (4.52- 6.70)	6.05 (4.56- 6.91)	0.77
	Month 6	5.40 (4.43- 6.61)	6.48 (4.70- 7.43)	0.35
Neutrophil Count (10^9/L)	Day 0	3.43 (2.49- 4.46)	3.32 (2.35- 4.14)	0.42
	Month 1	3.08 (2.42- 4.07)	3.22 (2.39- 3.86)	0.85
	Month 6	3.14 (2.53- 4.07)	3.52 (2.26- 4.44)	0.56
Lymphocyte Count (10^9/L)	Day 0	1.48 (0.98- 2.21)	1.90 (1.23- 2.36)	0.15
	Month 1	1.61 (1.07- 2.38)	1.89 (1.34- 2.47)	0.39
	Month 6	1.63 (0.99- 2.14)	1.90 (1.36- 2.37)	0.16
MCV (fL)	Day 0	91.10 (88.30- 95.40)	88.75 (86.10- 94.40)	0.15
	Month 1	91.60 (88.00- 94.90)	88.75 (85.85- 93.85)	0.09
	Month 6	91.30 (87.30- 93.20)	87.50 (84.20- 90.00)	0.02
Platelet Count (10^9/L)	Day 0	125.00 (90.00- 170.00)	114.00 (84.00- 194.00)	0.76
	Month 1	116.00 (89.00- 169.00)	129.50 (79.50- 186.00)	0.81
	Month 6	132.00 (96.00- 155.00)	138.00 (77.00- 182.00)	0.76
Sodium (mmol/L)	Day 0	141.00 (138.00- 143.00)	140.00 (138.00- 142.00)	0.23
	Month 1	141.00 (139.00- 143.00)	140.00 (138.00- 142.00)	0.26
	Month 6	139.00 (136.00- 141.00)	139.50 (136.00- 141.00)	0.74
Potassium (mmol/L)	Day 0	4.10 (3.80- 4.30)	4.20 (4.00- 4.50)	0.07
	Month 1	4.10 (3.90- 4.40)	4.30 (4.10- 4.60)	0.01
	Month 6	4.10 (3.80- 4.50)	4.30 (4.10- 4.50)	0.07
Urea (mmol/L)	Day 0	4.40 (3.60- 6.10)	4.00 (3.30- 5.50)	0.59
	Month 1	4.20 (3.30- 6.20)	4.30 (3.30- 5.80)	0.97
	Month 6	4.70 (3.60- 6.10)	4.40 (3.70- 5.80)	0.79
Creatinine (umol/L)	Day 0	73.00 (64.00- 87.00)	75.00 (64.00- 86.00)	0.89
	Month 1	76.00 (63.00- 89.00)	75.00 (63.00- 90.00)	0.95
	Month 6	78.00 (65.00- 88.00)	78.50 (71.00- 89.00)	0.70
Bilirubin (umol/L)	Day 0	16.00 (10.00- 25.00)	16.00 (10.00- 25.00)	0.54
	Month 1	14.00 (10.00- 21.00)	13.50 (10.00- 25.00)	0.73
	Month 6	15.00 (10.00- 25.00)	13.50 (10.00- 25.00)	0.66
Albumin (g/L)	Day 0	41.00 (38.00- 45.00)	41.00 (37.50- 43.00)	0.29
	Month 1	43.00 (40.00- 45.00)	41.00 (38.00- 44.00)	0.10
	Month 6	42.00 (38.00- 45.00)	42.00 (39.00- 44.00)	0.87
Alanine transaminase (IU/L)	Day 0	30.00 (20.00- 40.00)	28.00 (23.00- 48.00)	0.67
	Month 1	32.00 (24.00- 43.00)	33.00 (23.00- 47.00)	0.90
	Month 6	28.00 (21.00- 51.00)	30.00 (21.00- 43.00)	0.99
Aspartate Aminotransferase (IU/L)	Day 0	38.00 (28.00- 57.00)	39.00 (31.00- 47.00)	0.98
	Month 1	41.00 (28.00- 71.00)	35.50 (30.00- 49.00)	0.36
	Month 6	36.00 (24.00- 54.00)	33.00 (27.00- 43.00)	0.73
Alkaline Phosphatase (IU/L)	Day 0	81.00 (61.00- 110.00)	104.00 (81.00- 127.00)	0.01
	Month 1	84.00 (61.00- 113.00)	98.00 (79.00- 129.00)	0.10
	Month 6	83.00 (59.00- 109.00)	99.50 (74.00- 119.00)	0.07
γ-Glutamyl Transferase (IU/L)	Day 0	56.00 (31.00- 181.00)	82.00 (50.50- 178.50)	0.13
	Month 1	65.00 (34.00- 205.00)	90.00 (44.00- 195.00)	0.47
	Month 6	63.50 (41.00- 163.00)	95.50 (42.00- 160.50)	0.64
Amylase (U/L)	Day 0	78.50 (59.00- 97.00)	85.00 (56.50- 110.50)	0.45
	Month 1	77.50 (58.00- 94.50)	90.00 (64.00- 113.00)	0.09
	Month 6	75.50 (57.00- 89.00)	80.00 (55.00- 108.00)	0.43
C-Reactive Protein (mg/L)	Day 0	1.00 (0.50- 5.00)	2.00 (1.00- 6.00)	0.39
	Month 1	1.00 (0.50- 4.00)	1.00 (1.00- 5.00)	0.40
	Month 6	1.00 (1.00- 6.00)	2.00 (1.00- 4.00)	0.76

Table S2. A   Quality of Life Assessment using the SF-36 questionnaire (total population).

A.

Scale		Placebo (Mean[SD])	LcS (Mean[SD])	p value
Physical functioning	Day 0	67.66 (32.95)	58.43 (32.35)	0.25
	Month 1	75.17 (27.14)	63.00 (33.57)	0.13
	Month 6	68.83 (30.19)	65.86 (35.23)	0.73
Role limitations due to physical health	Day 0	60.71 (41.25)	53.91 (44.04)	0.52
	Month1	66.41 (41.93)	57.76 (46.34)	0.45
	Month 6	56.67 (45.93)	64.17 (41.88)	0.51
Role limitations due to emotional problems	Day 0	64.81 (42.87)	64.65 (42.44)	0.99
	Month 1	70.83 (42.96)	69.61 (41.33)	0.91
	Month 6	65.59 (42.59)	72.92 (37.33)	0.47
Energy/fatigue	Day 0	51.88 (27.29)	46.71 (24.34)	0.42
	Month 1	51.61 (25.21)	49.69 (24.16)	0.76
	Month 6	58.50 (22.40)	57.50 (20.96)	0.86
Emotional well-being	Day 0	69.14 (23.48)	62.16 (23.01)	0.21
	Month 1	73.33 (21.06)	64.97 (24.58)	0.14
	Month 6	75.03 (23.70)	64.39 (21.55)	0.07
Social functioning	Day 0	72.30 (31.06)	67.14 (29.56)	0.47
	Month 1	71.09 (27.02)	66.80 (32.32)	0.57
	Month 6	75.40 (31.04)	66.67 (31.20)	0.29
Pain	Day 0	66.94 (28.56)	69.49 (26.72)	0.69
	Month 1	74.14 (25.50)	64.63 (30.75)	0.18
	Month 6	68.17 (28.63)	68.05 (27.02)	0.99
General health	Day 0	49.38 (25.26)	48.65 (22.81)	0.9
	Month 1	50.34 (25.77)	43.83 (23.91)	0.32
	Month 6	49.00 (26.95)	49.33 (24.56)	0.96

Table S2. B   Quality of Life Assessment using the SF-36 questionnaire (alcoholic sub-population).

B.

Scale		Placebo (Mean[SD])	LcS (Mean[SD])	p value
Physical functioning	Day 0	64.64 (37.65)	66.25 (32.27)	0.9
	Month 1	74.06 (31.05)	57.00 (39.59)	0.19
	Month 6	67.50 (35.31)	63.21 (39.84)	0.76
Role limitations due to physical health	Day 0	62.50 (41.83)	56.67 (43.78)	0.71
	Month 1	69.12 (40.05)	48.08 (48.37)	0.2
	Month 6	70.31 (43.99)	59.38 (41.71)	0.48
Role limitations due to emotional problems	Day 0	68.63 (44.83)	77.78 (34.88)	0.53
	Month 1	74.51 (38.24)	62.50 (46.94)	0.43
	Month 6	75.00 (35.49)	72.92 (32.70)	0.86
Energy/fatigue	Day 0	58.00 (28.77)	47.22 (24.45)	0.25
	Month 1	59.12 (26.82)	49.69 (26.42)	0.32
	Month 6	64.06 (24.24)	54.38 (23.44)	0.26
Emotional well-being	Day 0	69.07 (26.90)	69.33 (19.26)	0.97
	Month 1	74.12 (22.68)	62.82 (21.90)	0.15
	Month 6	80.50 (21.51)	69.75 (17.03)	0.13
Social functioning	Day 0	74.31 (32.79)	71.32 (27.16)	0.77
	Month 1	73.53 (26.84)	66.41 (34.07)	0.51
	Month 6	76.56 (30.91)	66.96 (32.00)	0.41
Pain	Day 0	64.84 (31.89)	68.95 (28.31)	0.69
	Month 1	73.68 (27.70)	66.72 (33.81)	0.52
	Month 6	73.44 (30.62)	65.78 (29.44)	0.48
General health	Day 0	61.79 (23.58)	51.47 (21.56)	0.21
	Month 1	60.00 (25.63)	49.38 (24.42)	0.25
	Month 6	56.25 (29.75)	49.33 (21.78)	0.47

Table S2. C Quality of Life Assessment using the SF-36 questionnaire (non-alcoholic sub-population).

C.

Scale		Placebo (Mean[SD])	LcS (Mean[SD])	p value
Physical functioning	Day 0	70.00 (29.70)	51.84 (31.76)	0.08
	Month 1	76.54 (22.58)	69.00 (26.27)	0.43
	Month 6	70.36 (24.30)	68.33 (31.55)	0.85
Role limitations due to physical health	Day 0	59.21 (41.84)	51.47 (45.48)	0.6
	Month 1	63.33 (45.18)	65.63 (44.60)	0.89
	Month 6	41.07 (44.51)	69.64 (42.94)	0.1
Role limitations due to emotional problems	Day 0	61.40 (41.96)	53.70 (45.93)	0.6
	Month 1	66.67 (48.80)	75.93 (35.80)	0.53
	Month 6	55.56 (48.25)	72.92 (42.55)	0.3
Energy/fatigue	Day 0	46.47 (25.54)	46.18 (24.97)	0.97
	Month 1	42.50 (20.45)	49.69 (22.54)	0.37
	Month 6	52.14 (18.99)	61.07 (17.89)	0.21
Emotional well-being	Day 0	69.20 (21.29)	55.37 (24.67)	0.07
	Month 1	72.50 (19.91)	67.25 (27.68)	0.54
	Month 6	68.31 (25.38)	58.67 (24.83)	0.32
Social functioning	Day 0	70.39 (30.11)	63.19 (31.93)	0.48
	Month 1	68.33 (27.90)	67.19 (31.58)	0.92
	Month 6	74.17 (32.21)	66.35 (31.62)	0.52
Pain	Day 0	68.63 (26.33)	70.00 (25.85)	0.87
	Month 1	74.67 (23.71)	62.78 (28.62)	0.21
	Month 6	62.14 (25.96)	70.31 (25.13)	0.39
General health	Day 0	39.72 (22.65)	46.25 (24.11)	0.4
	Month 1	40.00 (22.36)	37.50 (22.51)	0.77
	Month 6	40.71 (21.47)	49.33 (27.83)	0.36

Table S3. A Number of abnormal neutrophil tests at baseline in placebo, LcS-treated and total patient groups.

	Patients with Abnormal Baseline Neutrophil Function		
	Placebo	LcS	Total
PBS-induced ROS	15	9	24
E. coli-induced ROS	12	13	25
fMLP-induced ROS	18	16	34
PMA-induced ROS	4	7	11
Phagocytosis	11	9	20

Table S3. B Number of patients in placebo, LcS-treated and total patient groups with abnormal neutrophil PBS-, *E.coli*, fMLP, PMA-induced Reactive Oxidant Species (ROS) production and phagocytosis.

Number of Abnormal Neutrophil Tests at Baseline	Placebo	LcS	Total
4	5	3	8
3	5	4	9
2	9	10	19
1	7	10	17

Table S4. Gastrointestinal Adverse Event Profile.

	Diarrhoea		Constipation		Abdominal discomfort		Bloating		p value
	Placebo	LcS	Placebo	LcS	Placebo	LcS	Placebo	LcS	(Placebo vs LcS)
Day 0-Day 14	2.38	6.82	2.38	2.27	11.9	4.25	7.14	6.82	>0.05
Day 14-Month 1	2.5	2.56	2.5	0	7.5	7.69	2.5	2.56	>0.05
Month 1-Month 3	0	5.56	3.23	0	0	2.78	3.23	2.78	>0.05
Month 3-Month 6	5.71	0	0	0	2.86	6.45	2.86	0	>0.05

Supplementary Methods

Analysis of neutrophil oxidative burst and phagocytosis

Phagoburst and Phagotest (BD Biosciences) kits were used to analyse oxidative burst and phagocytosis of neutrophils, respectively. Neutrophils isolated by Polymorphprep™ (Alere) were corrected to a concentration of 1x10^7/ml, and treated either with healthy or patient plasma for 90 minutes. Neutrophils were further stimulated with PBS (untreated), unlabelled opsonized bacteria (*E.coli*), labelled opsonized bacteria, N-formyl-Met-Leu-Phe (fMLP) and phorbol 12-myristate 13-acetate (PMA) for 20 minutes before analysis by flow cytometry using BD LSRFortessa™(BD Biosciences). Data from flow cytometry was analysed using FlowJo® 9.9.6 (BD). Neutrophil geometric mean fluorescent intensities (GMFI) were calculated from FSC/SSC high granulocyte populations expressing CD16 and CD11b.
